# Diisopropylamine dichloroacetate enhances radiosensitization in esophageal squamous cell carcinoma by increasing mitochondria-derived reactive oxygen species levels

**DOI:** 10.18632/oncotarget.11906

**Published:** 2016-09-08

**Authors:** Gaochao Dong, Qiang Chen, Feng Jiang, Decai Yu, Qixing Mao, Wenjie Xia, Run Shi, Jie Wang, Lin Xu

**Affiliations:** ^1^ Department of Thoracic Surgery, Jiangsu Key Laboratory of Molecular and Translational Cancer Research, Nanjing Medical University Affiliated Cancer Hospital, Cancer Institute of Jiangsu Province, Nanjing, Jiangsu, China; ^2^ Department of The Fourth Clinical College, Nanjing Medical University, Nanjing, Jiangsu, China; ^3^ Department of Thoracic Surgery, Xuzhou Centre Hospital, Xuzhou, Jiangsu, China; ^4^ Department of Hepatobiliary Surgery, The Affiliated Drum Tower Hospital of Nanjing University Medical College, Nanjing, Jiangsu, China

**Keywords:** diisopropylamine dichloroacetate, esophageal squamous cell carcinoma, ROS, radiosensitization

## Abstract

Radiotherapy is generally applied in the treatment of esophageal squamous cell carcinoma (ESCC). However, the radioresistance of ESCC still remains an obstacle for the curative effect of this treatment. We hypothesized that diisopropylamine dichloroacetate (DADA), an inhibitor of pyruvate dehydrogenase kinase (PDK), might enhance radiosensitizationin resistant ESCC. The clonogenic survival assay revealed that DADA sensitized ESCC cells to radiotherapy *in vitro*; furthermore, the combination of DADA and radiotherapy increased the expression of γ-H2AX, which is a hallmark of DNA double-strand breaks. Arrest at G2/M phase as well as the induction of apoptosis of ESCC cells were also observed in the cells treated with the combination of DADA and radiotherapy. Notably, xenograft tumor growth was significantly suppressed *in vivo* by combined radiotherapy and DADA administration. It has been proven that glycolysis is highly correlated with radioresistance, which could be reversed by the shift from glycolysis to mitochondrial oxidation. In our present study, we found that DADA could modulate oxidative phosphorylation as well as increase the intracellular levels of reactive oxygen species (ROS). Collectively, we concluded that DADA-induced intracellular ROS accumulation was identified as the key factor of radiotherapy sensitization of ESCC.

## INTRODUCTION

Esophageal carcinoma is the eighth most prevalent cancer and the sixth leading cause of cancer-related death worldwide, with a 5-year survival rate of < 20% [1–3]. Esophageal squamous cell carcinoma (ESCC), one of the main pathological subtypes of esophageal carcinoma, is predominantin East Asia and accounts for 95% of all Chinese esophageal carcinoma patients [4, 5]. Radiotherapy (RT) is recommended as the primary treatment modality for ESCC; however, the radioresistance of ESCC remains an obstacle for the effective treatment of ESCC [6]. Patients with ESCC who develop recurrent cancer tend to display a more aggressive phenotype because of the inherent ability of ESCC cells to become radioresistant [7]. Thus, developing a novel agent that can sensitize cancer cells to radiation may ultimately improve the effectiveness of radiation treatment and minimize the risk of recurrence.

Enhanced glycolysis is a common trait of many types of human cancers [8]. To produce energy and maintain proliferation, cancer cells show an enhanced reliance on glycolysis even in the presence of oxygen (Warburg effect) [9]. Consistent with many types of solid tumors, ESCC is highly glycolytic, producing large amounts of lactic acid as a metabolic by-product [10]. Accumulated evidence supports the notion that glycolysis is associated with drug resistance and radioresistance in cancer therapy [11–13]. In this vein, switching cancer cells from glycolysis to oxidative phosphorylation may increase tumor cell sensitivity to RT [14, 15]. Dichloroacetate (DCA), an inhibitor of pyruvate dehydrogenase kinase (PDK), leads to the reactivation of pyruvate dehydrogenase (PDH) and shifts glucose metabolism from glycolysis to mitochondrial oxidation [16]. Preclinical trials on DCA have shown that DCA could enhance the radiosensitivity of several tumor types when combined with RT [14, 17, 18]. However, it has not been permitted for use as an anti-cancer drug in the clinic. Therefore, it is urgent to discover a safer and more potent drug that could sensitize cancer cells to RT.

Diisopropylamine dichloroacetate (DADA) is the active component of pangamic acid and has been commercially available for over 50 years for the treatment of chronic liver disease [19]. It has been previously reported that DADA is a safe inhibitor of pyruvate dehydrogenase kinase 4 (PDK4), which is a PDK subtype [20]. We thus hypothesized that DADA could enhance radiosensitization in esophageal squamous cell carcinoma by modulating the metabolism of the cancer cells.

In this study, we demonstrated that DADA, which is a more effective anti-cancer drug than DCA in ESCC, could sensitize ESCC cells to radiotherapy both *in vitro* and *in vivo*. Increasing levels of mitochondria-derived reactive oxygen species (ROS) could be the potential mechanism through which DADA enhances the radiotherapy sensitivity of ESCC cell lines.

## RESULTS

### By comparison with DCA, DADA exhibited more anti-tumor effects in ESCC cells

**Figure 1:**
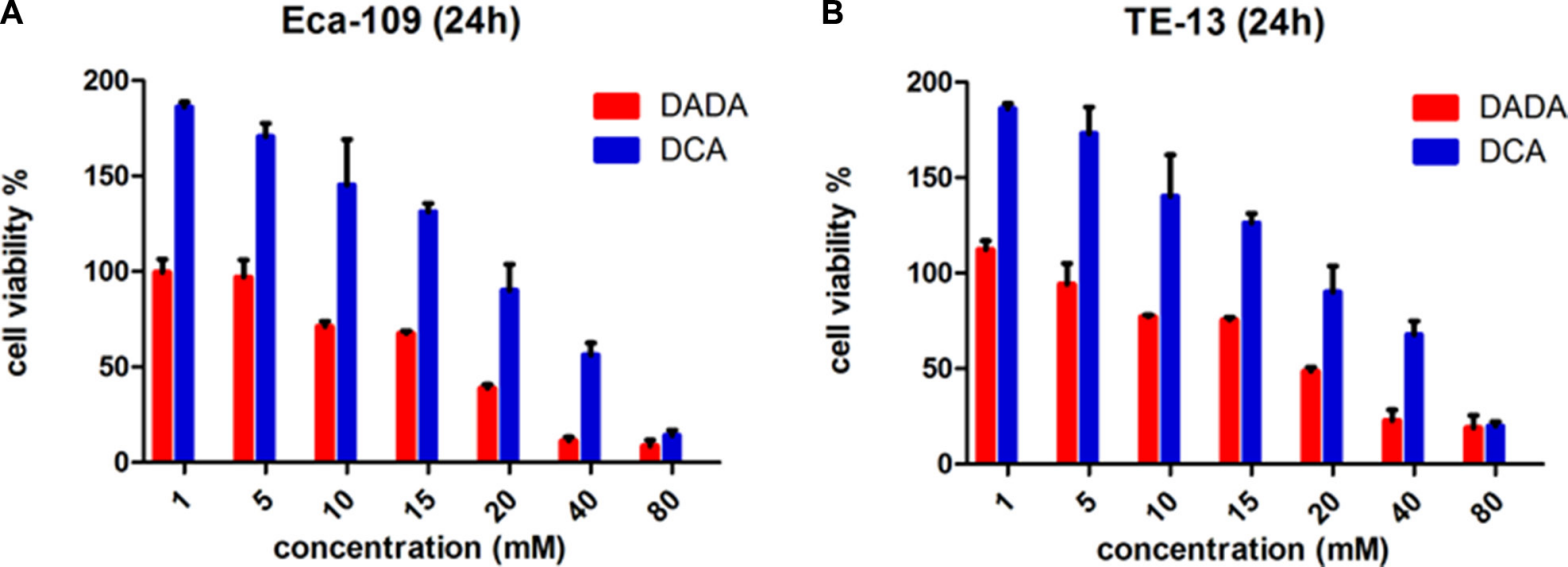
Toxicity of DADA and DCA on human esophageal squamous cell carcinoma cell lines The ESCC cell lines Eca-109 (**A**) and TE-13 (**B**) were seeded in 96-well plates in triplicate and treated with various concentrations of DADA and DCA for 24 h. Cell viability was determined by the CCK8 assay.

We first assessed the anti-tumor effect of DADA and DCA in the two ESCC cell lines Eca109 and TE-13. We evaluated the viability of ESCC cells after a 24-h incubation with increasing concentrations of DADA and DCA. At 24 h, the IC_50_ of DADA in Eca-109 cells was18 mM. However, the IC_50_ of DCA in Eca-109 cells was 32 mM (Figure 1A). The viability of TE-13 cells after a 24-h incubation with both agents was also evaluated. The IC_50_ values of DADA and DCA in TE-13 cells were 22 and 33 mM, respectively (Figure 1B). These results suggested that DADA exerted more inhibitionof proliferation in ESCC cell lines than DCA.

The sub-toxic concentrations of DADA in Eca-109 and TE-13 cells were 10 mM and 8 mM, respectively; neither of these concentrations could significantly inhibit the proliferation of the two cell lines. These concentrations were adopted for the subsequent experiments of the two cell lines.

### DADA sensitized ESCC cells to radiotherapy *in vitro*

**Figure 2:**
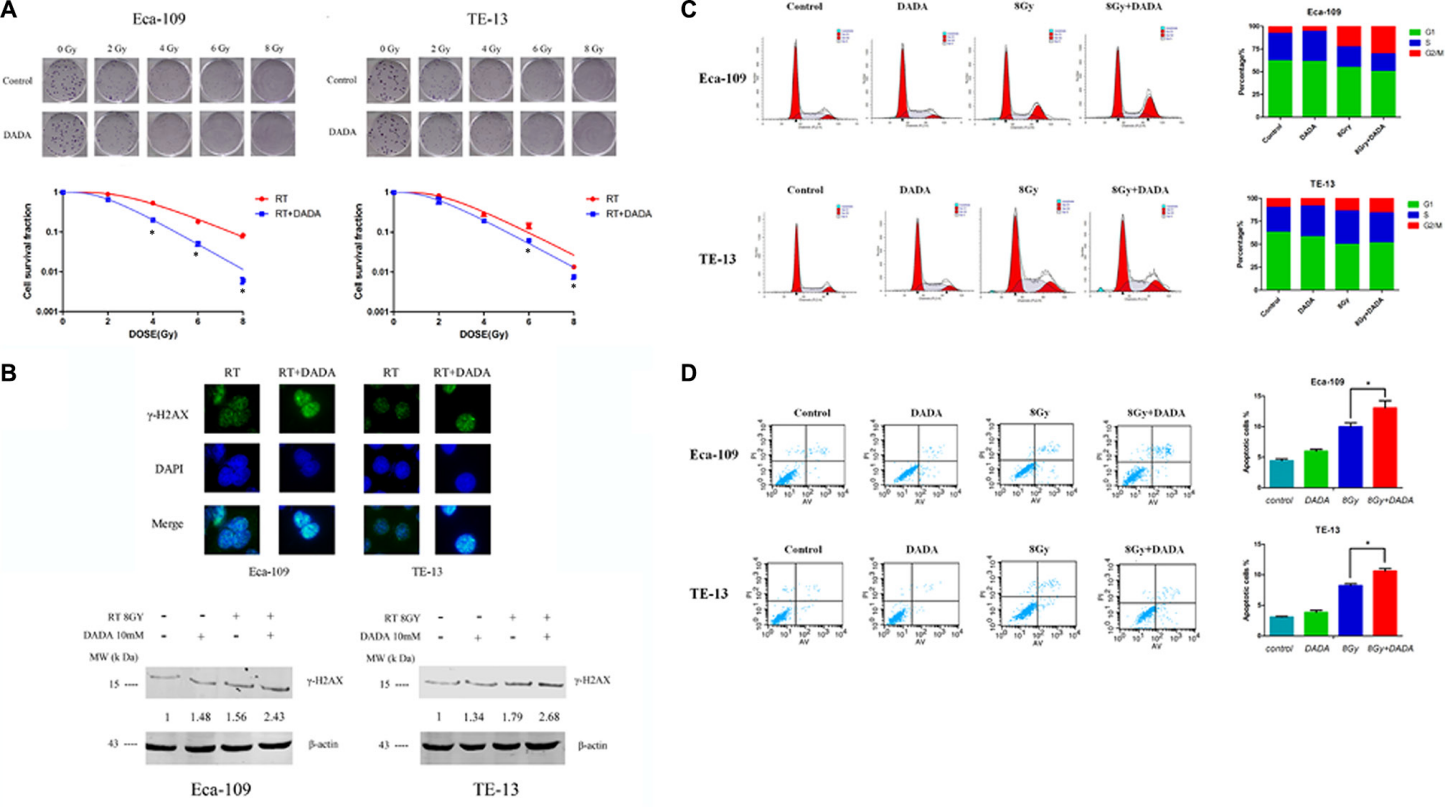
DADA sensitized ESCC cells to radiotherapy *in vitro* (**A**) The survival curves of ESCC. The clonogenic survival assay was used to assess the radiosensitization of DADA on Eca-109 and TE-13 cells. The cells were treated with 10 mM or 8 mM, respectively, and ionizing radiation as illustrated followed by harvesting after incubation for 10–14 d. (**B**) DADA increased the number of DNA double-strand breaks induced by RT. Eca-109 and TE-13 cells were treated with eitherX-rays alone or in combination with DADA. After incubation for the appropriate time, the cells were fixed for the immunofluorescence detection of γ-H2AX (green). DAPI was used for nuclear staining. Eca-109 and TE-13 cells were treated with either X-rays alone or in combinationwith DADA and subjected to Western blot analysis of the DNA double-strand break-related protein γ-H2AX. (**C**) Cell cycle distribution after different treatments of ESCC cell lines. Eca-109 and TE-13 cells were treated with DADA, X-rays or the combination treatment and then collected for analysis of cell cycle distribution by flow cytometry. (**D**) Apoptosis percentage in different treatments of ESCC cell lines. Cells were seeded into 6-well plates at the adequate concentration; incubated with DADA, X-rays or the combination treatment and then harvested after incubation for forty-eight hours. The cells were examined by flow cytometry for apoptosis after staining with annexin-V-FITC/PI. The data are presented as the mean ± SD. * indicated statistical significance versus the control group (*p* < 0.05).

ESCC cells were treated with DADA for 24 h. The survival fraction (SF) was calculated from the number of clones after radiation of 0, 2, 4, 6 and 8Gy. In Eca-109 cells, the SF of the RT+DADA group decreased significantly with each increasing dose of radiation compared with the RT group (Figure 2A). However, compared to the RT group, the SF of the RT+DADA group just decreased a little in various dose of radiation in TE-13 cells. The single-hit multi-target was adopted to generate the survival curves of the ESCC cell lines. The data changed markedly when comparing the RT+DADA and RT groups (Table 1). The survival fraction after 2Gy (SF2) and the values of D_0_ and D_q_ in Eca-109 cells were 0.89, 1.86Gy and 1.41Gy, respectively, in the control group and 0.66, 1.36Gy and 0.84Gy in the combination group, whereas in TE-13 cells the corresponding values were 0.81, 1.52Gy and 1.08Gy in the control group and 0.62, 1.44Gy and 0.77Gy in the combination group. The sensitization enhancement ratio (SER) of the Eca-109 and TE-13 cells were 1.37 and 1.06, respectively. These data indicated that DADA significantly increased cell death in irradiated ESCC cells. To further explore the radiosensitization of DADA on ESCC cells, we detected γ-H2AX, a hallmark of DNA double strand breaks, after treatment with radiotherapy (Figure 2B). Immunofluorescence detection of γ-H2AX (green) was performed 4 hours after radiotherapy (8 Gy). For TE-13 cells, the combination of DADA with radiotherapy led to a significant increase in γ-H2AX compared with radiotherapy alone. Compared with the TE-13 cells, Eca-109 cells could also be radiosensitized by the combined treatment, as indicated by significant increase in the number of positive γ-H2AX foci.

**Table 1:** Radiosensitization effects of DADA on ESCC cells *in vitro*

	D_0_	D_q_	SF_2_	SER
Eca-109	1.86	1.41	0.89	
Eca-109+DPDA	1.36	0.84	0.66	1.37
Te-13	1.52	1.08	0.81	
Te-13+DPDA	1.44	0.77	0.62	1.06

D_0_, lethal radiation dose; D_q_, quasi-threshold dose; SF_2_, fraction of cells surviving after 2 Gy radiation; SER, radiosensitization radio (sensitization enhancement ratio), calculated as D_0_ for the control group divided by D_0_ for the treatment group.

We also took advantage of Western blotting to measure the protein levels of γ-H2AX after radiotherapy (8 Gy), DADA treatment and the combination treatment (Figure 2B). For Eca-109 cells, both radiotherapy and DADA treatment alone could increase the production of γ-H2AX slightly (1.48- and 1.56-fold, respectively), whereas the combination induced significant γ-H2AX production (2.43-fold). Similar results were also observed in the TE-13 cells, with 1.37-, 1.79- and 2.68-fold increases after radiotherapy, DADA and combination therapy, respectively. Based on the above results, the combined treatments significantly increased γ-H2AX production, which is a hallmark of DNA damage.

Cell cycle analysis by flow cytometry showed that DADA had a differential effect on the proportion of ESCC cells in G2/M phase (Figure 2C). In Eca-109 cells, the proportion of cells in G2/M phase was significantly higher in the combination group compared with the RT and DADA groups, which indicated that DADA markedly influences the distribution of cell cycle in Eca-109 cells. However, in TE-13 cells, the proportion of cells in G2/M phase was not significantly changed in the combination group, which indicated that the effect of DADA on G2/M phase arrest was not remarkable.

Next we analyzed radiation-induced apoptosis in ESCC cells after treatment with DADA. The concentration of DADA administered to the Eca-109 and TE-13 cell lines was 10 mM and 8 mM, respectively. The difference among the groups receiving either combination individual treatments was statistically significant (*p <* 0.05) in both Eca-109 and TE-13 cells (Figure 2D). The apoptosis of both cell lines was markedly increased when DADA was administered in combination with radiation.

### DADA radiosensitized Eca-109 *in vivo* xenograft tumor

**Figure 3:**
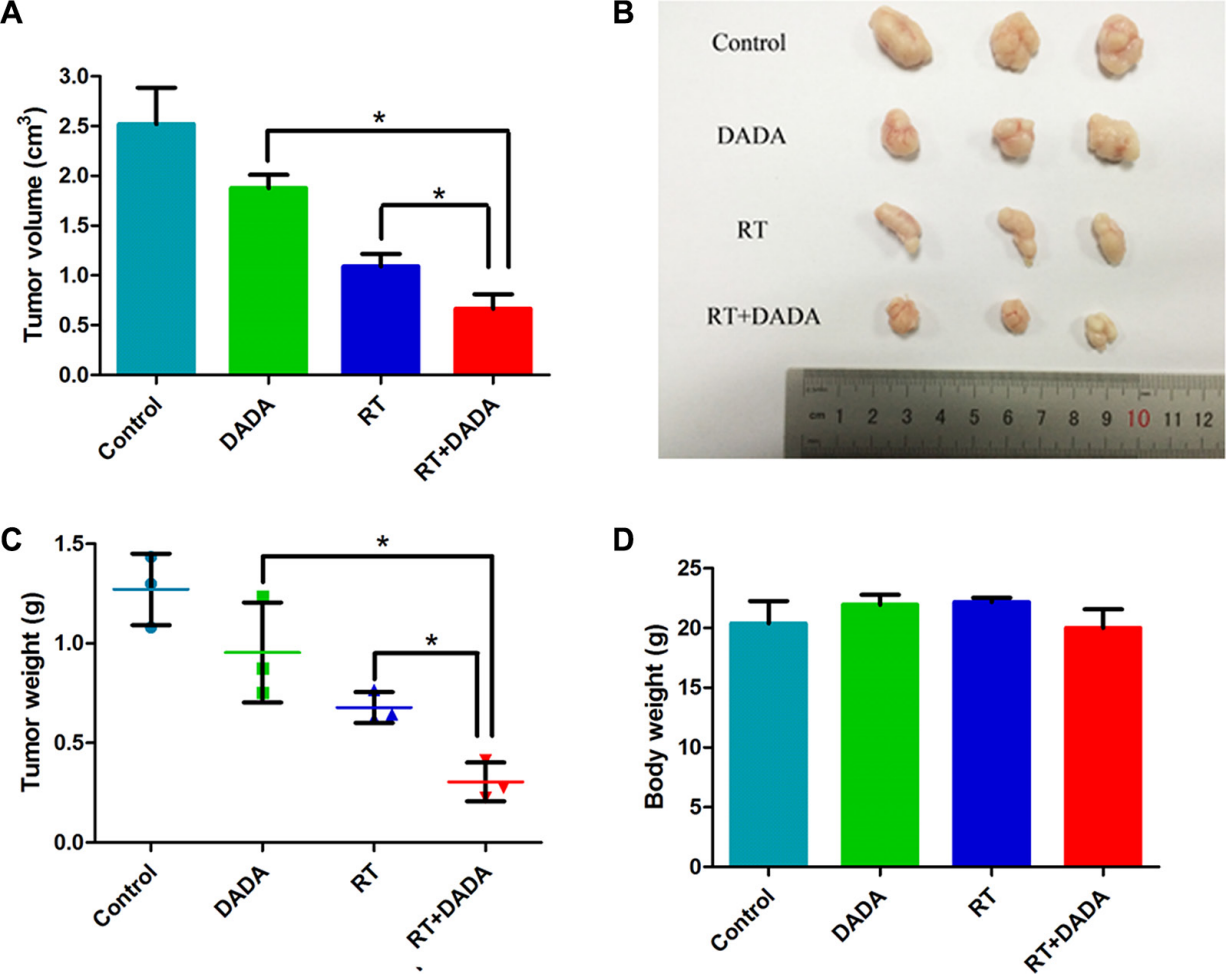
DADA sensitized ESCC cells to radiotherapy *in vivo*. (**A**–**B**) The tumor volume of each group was measured at the end of the observation. (**C**) The tumor weight was measured at the end of the observation. (**D**) The body weight of the mice subjected to the different treatments was measured at the end of the observation. The data are presented as the mean ± SD. *indicated statistical significance versus the control group (*p* < 0.05).

Mice bearing Eca-109 cell xenograft tumors were utilized to determine the radiosensitizing activity of DADA *in vivo*. As shown in Figure 3A and 3B, both the single DADA and radiotherapy treatments alone reduced the tumor volume growth compared to the control group. Notably, the combination of DADA and radiotherapy produced more tumor volume regression (*P <* 0.05) versus either radiotherapy or DADA treatment alone. As expected, the tumor weight was smaller in the combination group compared with either single treatment group (Figure 3C). Body weight was not significantly different in each group (Figure 3D). The results demonstrated that DADA could suppress the growth of xenograft tumors *in vivo* when combined with radiotherapy.

### DADA increased ROS levels in irradiated ESCC cells and increased the basal oxygen consumption rate (OCR) of ESCC cells

In a previous study, it has been proven that DADA suppresses the activity of pyruvate dehydrogenase kinase 4, leading to reactivation of mitochondria [20]. We hypothesized that DADA could increase the oxidative intracellular stress and synergistically reverse glycolysis.

**Figure 4:**
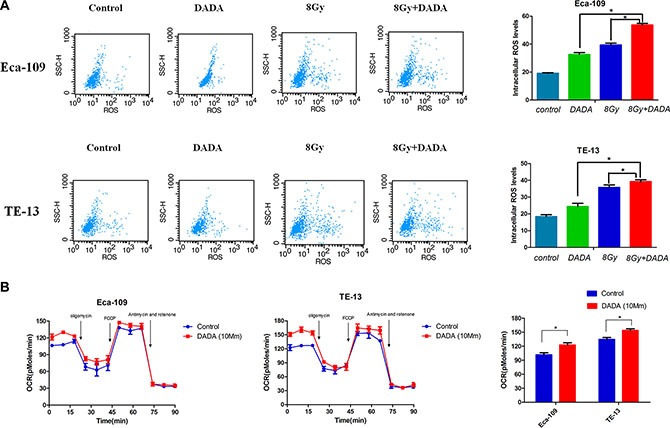
The combination of DADA and radiotherapy increased the oxygen consumption rate (OCR), resulting in increased intracellular levels of ROS in ESCC cell lines (**A**) ESCC cell lines were subjected to DADA, X-rays or the combination treatments and harvested for analysis of their ROS levels by flow cytometry. (**B**) Eca-109 and TE-13 cells were treated with DADA. The bioenergetic profiles of these cells were measured using sequential injection of oligomycin, FCCP, and a mixture of antimycin and rotenone.* indicated statistical significance versus control cells (*p* < 0.05).

In Eca-109 cells, relative ROS levels of 170% and 205% were measured after individual treatment with either DADA or RT, respectively, whereas in TE-13 cells, the relative ROS levels were 132% and 194%, respectively (Figure 4A). When treating with the DADA and RT combination, the levels of ROS increased significantly compared with either single treatment or in Eca-109 cells, which indicated that DADA could enhance the intracellular levels of ROS when combined with radiation. However, only a slight change of ROS levels was observed in TE-13 cells administered the combination treatment.

The oxygen consumption rate (OCR), which is positively related to mitochondrial oxidation, was measured using an XF Extracellular Flux Analyzer. After a 24-hour treatment with DADA, the basal OCR of Eca-109 and TE-13 cells increased to 23 pMoles/min and 25 pMoles/min, respectively (Figure 4B). While the maximal respiratory capacity of tumors cells was not obviously affected by DADA treatment. The experimental results indicated that DADA could modify oxidative phosphorylation and, as a result, increase the intracellular levels of ROS.

## DISCUSSION

In the present study, we have shown that DADA inhibited the growth of esophageal cancer cells *in vitro*. By using clone formation assay, which is one of the most reliable methods to detect cell survival and is the gold standard for detecting the radiosensitivity of cells, DADA sensitized ESCC cells to radiotherapy at a concentration of low toxicity. Radiosensitization by DADA in ESCC was associated with the arrest at the G2/M phase as well as the induction of apoptosis. Additionally, DADA and RT synergistically induced the production of γ-H2AX, a hallmark of DNA damage. Additionally, the intracellular levels of ROS were increased after the combination treatment compared with the levels of both single treatments. *In vivo* experiments also indicated that the combination of DADA and radiotherapy significantly suppressed tumor volumes. Above all, these findings provided a clue that the conversion from glycolysis to mitochondrial oxidationmay serve as an attractive approach to sensitize these malignant cells to RT.

Ionizing radiation (IR) is one of the main methods used in the management of cancer [7]. The important physical stimulus produced by IR is high levels of ROS [21]. IR plays roles in the treatment of cancer both directly and indirectly [22]. The radiation energy and the ROS produced from intercellular water caused a breakdown of double-stranded DNA and directly damaged cellular proteins. The indirect effect is the secondary response involved in gene expression and cell signaling by the second messenger ROS. However, it has been reported that radiotherapy acts as a double-edged sword [23]. Radiotherapy has been proven to be linked with the activation and stabilization of hypoxia-inducible factor-1 (HIF-1), a transcription factor that activates the transcription of numerous genes involved in angiogenesis, proliferation and glycolytic metabolism. HIF-1 activates the glucose transporter GLUT1 and pyruvate kinase M2 (PKM2) both of which play important roles in glycolysis [24]. Additionally, HIF-1 activates pyruvate dehydrogenase kinases (PDKs), which inactivate pyruvate dehydrogenase and prevent pyruvate from entering the TCA cycle. Stabilization of HIF-1 induced by IR enhances glycolysis in cancer cells [25].

A number of studies has demonstrated that glycolysis is highly correlated with radioresistance [14, 15, 26–30]. Tumor cells undergoing glycolysis not only produce large number of ATP molecules, but they could also produce many macromolecules such as pyruvate, lactate, glutathione and NAD(P), all of which compose an intracellular redox buffer network to effectively scavenge ROS and reduce the efficacy of RT as a consequence. Therefore, modulating glycolysis in cells to sensitize them to RT could be an effective approach in treating cancer.

Meng and his colleagues demonstrated that knockdown of PKM2 expression using pshRNA-PKM2 effectively enhanced the radiosensitivity of NSCLC cell lines and xenografts [15]. 3-Methyl pyruvate (MP), a membrane-permeable pyruvate derivative that is capable of activating mitochondrial energy metabolism, induces radiosensitization of A549 cells via the production of excess mitochondria-derived ROS [31]. Knockdown of hexokinase 2 (HK2), the upregulation of which elevates aerobic glycolysis, effectively enhanced the sensitivity of latent membrane protein 1 (LMP1)-overexpressing nasopharyngeal carcinoma cells to irradiation [32]. DCA, an inhibitor of PDKs, can effectively sensitize glioblastoma (GBM) cells to RT by modulating the metabolic state of tumor cells [33]. All of these studies confirmed that reversing glycolysis effectively sensitized cancer cells to radiotherapy.

To our knowledge, this is the first application of DADA as a radiosensitization agent. We proved that DADA modulated mitochondrial oxidation and intracellular levels of ROS, which rendered ESCC cells sensitive to radiotherapy. In future studies, we should explore the role of ROS by-products produced by DADA in radiosensitization. The increasing levels of ROS may act on a single transduction mechanism to mediate sensitization. As a radiosensitization agent, the safety of DADA should be affirmed. If all these questions could be resolved, DADA would be very beneficial in clinical applications.

## MATERIALS AND METHODS

### Cell lines and reagents

The human esophageal squamous cell carcinoma cell lines Eca-109 and TE-13 were purchased from the Chinese Academy of Science (Shanghai, China). The cells were maintained in DMEM (Dulbecco's modified Eagle's medium, GIBCO) with 10% FBS (fetal bovine serum, GIBCO) at 37°C in a humidified atmosphere with 5% CO_2_. DCA was obtained from Sigma (CAS: 79-43-6) and DADA was purchased from the company of Sunlidabio (Nanjing, China, CAS: 660-27-5).

### Drug treatment and irradiation conditions

DCA and DADA stock solutions (160 mM) were diluted in DMEM at the desired concentration for the *in vitro* experiments. X-ray radiation was delivered by a 6 MV linear accelerator (Elekta, Stockholm, Sweden) at a dose rate of 250 cGy/min with a source-to-target distance of 100 cm.

### Cell viability assay

Cells seeded in 96-well plates overnight were treated with either DCA or DADA at various concentrations (0, 1, 5, 10, 15, 20, 40, and 80 mM). After 24 hours, the medium was replaced with 10 μl of CCK-8 solution (Cell Counting Kit-8, KeyGen, Nanjing, China). The absorbance of the resulting formazan was determined at 450 nm after a 1-hour incubation. The viability of cells was calculated as follows: viability = (OD_test group_ – OD _blank group_)/(OD _control group_ – OD _blank group_)×100%. All of the experiments were repeated in triplicate.

### Clonogenic survival assay

Approximately 5 × 10^5^ esophageal cancer cells were plated into sterile T25 flasks and allowed to adhere overnight. The following day, cells were treated with either 10 mM DADA or DMEM (control). After 24 hours, flasks were irradiated with a total of dose of 2, 4, 6,or 8 Gy or left unirradiated as a control. Immediately after irradiation, cells were trypsinized, serially diluted, replanted onto 10-cm dishes, and incubated for 14 days. Next, colonies were stained with 0.2% crystal violet and counted. The surviving fraction (SF) was calculated relative to the unirradiated (control) cells. Each experiment was performed in triplicate, and the mean SF for each set of three experiments was calculated.

### Flowcytometry for cell cycle and apoptosis

Eca109 and TE-13 cells were seeded in a 6-well plate at 2 × 10^5^ cells/ml and harvested after 24 h treatment with DADA and/or 8Gy X-rays as appropriate. The cells were washed with ice-cold PBS and fixed with ice-cold 70% ethanol. After storage at −20°C, the cells were washed with PBS and resuspended in 0.5 ml propidium iodide (PI)/RNase staining buffer (BD Bioscience, USA) for 30 minutes at room temperature in the dark. The assessment of cell cycle distribution was performed using the FACS Calibur flow cytometer (BD Bioscience, USA). The results shown arerepresentative of at least three separate experiments.

The annexin V-FITC and PI binding assay (Keygen Biotech, Nanjing, China) was performed to assess the apoptosis and necrosis of cells *in vitro*. Approximately 2 × 10^5^ cells/ml of either Eca-109 or TE-13 cells were seeded in a 6-well plateovernight. After a 24-hour incubation with either DADA or DMEM (10% FBS), the cells were irradiated to the dose of 8Gy. Immediately, the medium was replaced with DMEM (10% FBS). After a 24-hour incubation, the cells were trypsinized, washed in PBS and resuspended in 100 μl of binding buffer. Before analysis, the cells were cultured in the dark for 15 minutes with 10 μl ofannexin V-FITC and 10 ul PI followed by 400 μl of binding buffer. Apoptosis and necrosis were determined using the FACS Calibur flowcytometer (BD Bioscience, USA). Each experiment was performed in triplicate.

### Immunofluorescence

DNA double strand breaks (DSBs) were detected through immunofluorescence of phosphor-H2AX. The appropriate cells were seeded on coverslips treated with either DADA alone or combination with X-rays. After a 4-hour exposure to X rays, cells were fixed in methanol for 30 min at room temperature and permeabilized with 0.1% Triton X-100 for 10 min at 4°C. Cells were incubated with an antibody against phospho-H2AX (Ser139) (Abcam, ab2893) after blocking with 4% BSA (dissolved in PBS) for 30 min at room temperature. The following day, cells were stained with fluorescein (FITC)-conjugated goat anti-mouse IgG (KeyGEN) for 1 hour at room temperature. DAPI was used to stain the cellnuclei. A Zeiss fluorescence microscope (Axio Vert. A1) was used to examined the immunoreactive foci for γ-H2AX.

### Western blotting assay

Cells were seeded in 6-well plates and treated with DADA and/or 8Gy X-rays as appropriate. The cells were washed with ice-cold PBS, lysed with RIPA buffer (KeyGEN) and centrifuged (14000 rpm, 10 min, 4°C). The protein concentration was measured using the BCA Assay Kit (KeyGEN). Western blot analysis was performed as previously described used the following antibodies: γ-H2AX (Abcam, ab2893) and β-actin (Abcam, ab8227).

### Reactive oxygen species (ROS) measurement

A Reactive Oxygen Species Assay kit (KeyGEN) was used to detect the intracellular levels of ROS. Briefly, 2 × 10^5^ cells/ml of either Eca109 or TE-13 cells were seeded in a 6-well plate andtreated with DADA and/or 8Gy X-rays as appropriate. DCFH-DA was dissolved in serum-free medium at the appropriate concentration (10 mmol/L). Cells were washed three times with PBS,and 1 mL of DCFH-DA solution was added. After a 30-min incubation at 37°C, cells were harvested by trypsinization and resuspended in serum-free medium. The level of ROS was detected using the FACS Calibur flow cytometer (BD Bioscience, USA). Each experiment was performed in triplicate.

### Extracellular flux assay

The XF Extracellular Flux Analyzer (Seahorse Bioscience) was used to measure the oxygen consumption rate (OCR). Cells were seeded (8000 cells/well) in 96-well plates from Seahorse Biosciences and allowed to adhere overnight in culture media. The following day, cells were treated as indicated. On the day of the assay, cells were washed and modified DMEM media were added. The cartridge was loaded to dispense 3 metabolic inhibitors sequentially at specific time points: oligomycin (1 mmol/L), FCCP (1 mmol/L), and a combination of rotenone and antimycin (both 0.5 mmol/L). Oligomycin, an inhibitor of ATP synthase, is used to measure the rate of proton leak in the mitochondrial membrane. FCCP, a protonophore and uncoupler of mitochondrial oxidative phosphorylation, is used to measure the maximum respiration rate. The combination of rotenone and antimycin inhibits the transfer of electrons from iron-sulfur centers to ubiquinone, thus inhibiting oxidative phosphorylation.

### Xenograft tumor radiosensitivity studies

Animal experiments were approved by the Ethics Committee of Nanjing Cancer Hospital. Five- to six-week-old male BALB/C nude mice were provided by the Nanjing Medical University Animal Center. Approximately 1 × 10^6^ Eca-109 cells were suspended in 0.1 mL PBS and injected s.c. into one site of the right leg of nude mice. Tumors were allowed to grow for 10 days before treatment. Mice were randomized into the following four groups: (a) vehicle (PBS) alone; (b) DADA alone (50 mg/kg as a 7-day continuous infusion); (c) a single dose of 8Gy IR; or (d) DADA plus IR (a single fraction of 8Gy IR after DADA treatment). Body weight and tumor volume were measured every 2 days in the mice, and the tumor volumes were measured as length × width^2^ × 0.5. The first day of treatment was designated as day 0, and observation continued until day 20. At the end of observation, mice were euthanized. The tumor weight, tumor volume and body weight were recorded for further analysis.

### Statistical analysis

All of the experiments were representative of at least three replicates, and the data were expressed as the mean ± SD. Significant differences between the groups were analyzed using Student's *t-test*. Statistical analysis was performed using SPSS (version 17.0; SPSS, Inc.), and *p <* 0.05 was considered to be statistically significant.
